# Supplementary material for: Circ-CPSF1 Worsens Radiation-Induced Oxidative Stress Injury in Caenorhabditis elegans
Source: Biomolecules. 2023 Jan 4;13(1):102. doi: 10.3390/biom13010102 (PMC9856148; doi:10.3390/biom13010102)
Supplement: Supplementary file 1 [file biomolecules-13-00102-s001.zip › biomolecules-2014137-supplementary.pdf]

Table S1. Real-time qPCR primers of target circRNAs.

Gene	Forward Primer	Reverse Primer
circ-CPSF1	ATCCGATTCCTGGACACC	TCAGCCAATGGGAAATGC
circ-DYN1	CACCCATCTGGACCTTCAC	TGGTGCATCCCAACTCAA
circ-Y38F2AR.12	GGGTCTACTTGCTGGAGGA	GTTTCGATTTGCGATTCTGT
circ-CNT2	GTCAACTGGCCGCTCAAAT	TTGTTGGCCTTCGTCTCG
circ-Y48A5A.1	TTGGAGCAAGTTTGGAGTTATT	ATTCGCCTTCGTCGGATT
circ-Mig10	CACCGGCTCTTTCTTATCACC	GTTCATCCAACTGCCAATCT
circ-MAM3	ATCAGTGGGTCAACTACGATGGAG	GGAAGAGTTTGGATGGGATT
circ-Zip1	GACGGCGGTGGATAAGAA	TCCGAGAAATCGACGAAA
circ- Y51H1A.2	TCGGAGCCAACCAATGTC	CTCCAGCCAACTCGTGAAG
circ-DYS1	TGAGGGAGAAGAGCAGAAAAATC	AGCTCATCAAATAGCATAGCACC

Table S2. Real-time qPCR primers of target genes.

Gene	Forward Primer	Reverse Primer
act-2	CCCACTCAATCCAAAGGCTA	GGGACTGTGTGGGAACACC
sod-1	CGAGGGAGTCGGAGACAAGG	GTAGTAGGAGTAGGAACAAC
sod-3	TTGAAGATCGCCACCTGTGCAAAC	ATGGACATAGTCTGGGCGGACATT
ctl-2	GACAATCAGCAACATGCTCC	CTGGCACATTCTCTCCCGAG
ctl-3	CACGCATTACCATCGCCTTG	CAACGAGCCGATCTCTCTCC
hus-1	GTTCTGCCCGGCGGACACTG	TGTGTCCAATTGACGGCCTGGA
clk-2	CCGAATCCTCCATCTTCAAA	CAATTGTTGCGCCATTACAC
mrt-2	TCAATCCGGCGTTCTTCTCG	AAACCCGGCGAACTTGATCT
cep-1	TGTCCAGAAAATGATAGACGGAGT	GCATCGGAAATCTTTGGCGT
egl-1	CACCTTTGCCTCAACCTC	TTGGAGCCGATCTCGTAG
ced-13	ACGGTGTTTGAGTTGCAAGC	GTCGTACAAGCGTGATGGAT
ced-9	CGAACGATGGCGACTGGCGA	CGGTTGCACTCCACACGGCA
ced-4	TCTACACGGCCGAGCTGGAT	TCGTCTTCGCTTTTTAGCATCAGCA
ced-3	TCGACGGAGTTCCTGCATTT	GCTTGGCTCGGCTTCTTTCT

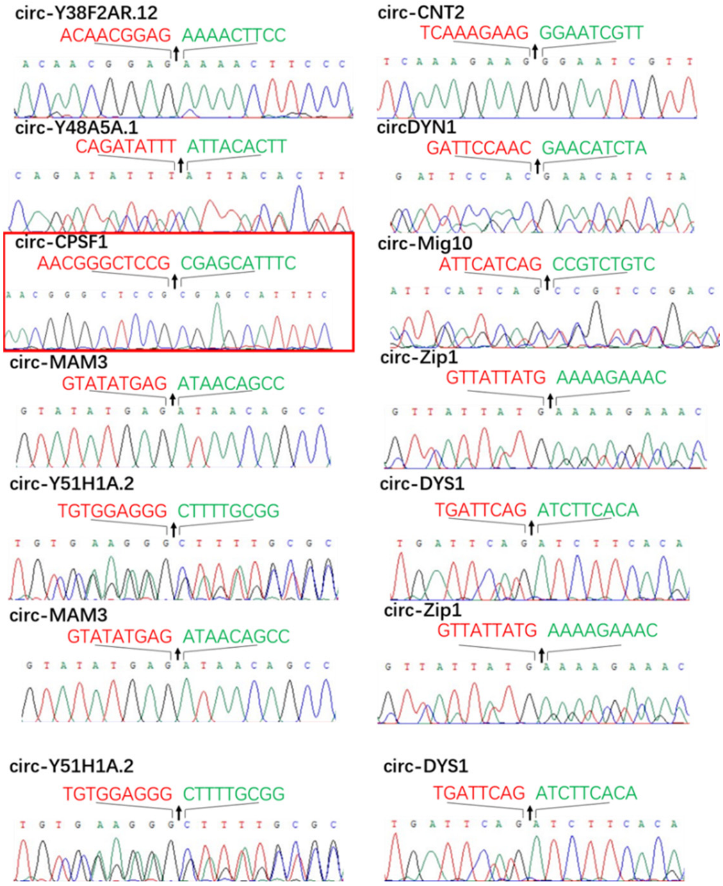

Figure S1. Sanger sequencing was used to certify the existence of spliced junctions of 10 circRNAs.

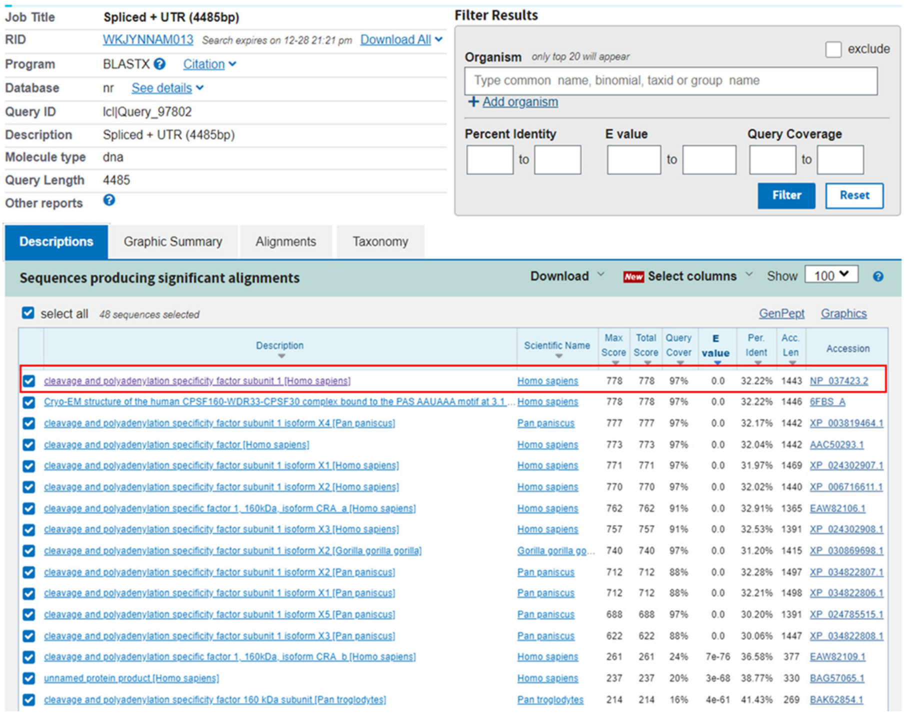

Description	Scientific Name	Max Score	Total Score	Query Cover	E value	Per. Ident	Acc. Len	Accession
cleavage and polyadenylation specificity factor subunit 1 [Homo sapiens]	Homo sapiens	778	778	97%	0.0	32.22%	1443	NP_037423.2
Cryo-EM structure of the human CPSF160-WDR33-CPSF30 complex bound to the PAS AAUAAA motif at 3.1 ...	Homo sapiens	778	778	97%	0.0	32.22%	1446	6FBS_A
cleavage and polyadenylation specificity factor subunit 1 isoform X4 [Pan paniscus]	Pan paniscus	777	777	97%	0.0	32.17%	1442	XP_003819464.1
cleavage and polyadenylation specificity factor [Homo sapiens]	Homo sapiens	773	773	97%	0.0	32.04%	1442	AAC50293.1
cleavage and polyadenylation specificity factor subunit 1 isoform X1 [Homo sapiens]	Homo sapiens	771	771	97%	0.0	31.97%	1469	XP_024302907.1
cleavage and polyadenylation specificity factor subunit 1 isoform X2 [Homo sapiens]	Homo sapiens	770	770	97%	0.0	32.02%	1440	XP_006716611.1
cleavage and polyadenylation specific factor 1, 160kDa, isoform CRA_a [Homo sapiens]	Homo sapiens	762	762	91%	0.0	32.91%	1365	EAW82106.1
cleavage and polyadenylation specificity factor subunit 1 isoform X3 [Homo sapiens]	Homo sapiens	757	757	91%	0.0	32.53%	1391	XP_024302908.1
cleavage and polyadenylation specificity factor subunit 1 isoform X2 [Gorilla gorilla gorilla]	Gorilla gorilla go...	740	740	97%	0.0	31.20%	1415	XP_030869698.1
cleavage and polyadenylation specificity factor subunit 1 isoform X2 [Pan paniscus]	Pan paniscus	712	712	88%	0.0	32.28%	1497	XP_034822807.1
cleavage and polyadenylation specificity factor subunit 1 isoform X1 [Pan paniscus]	Pan paniscus	712	712	88%	0.0	32.21%	1498	XP_034822806.1
cleavage and polyadenylation specificity factor subunit 1 isoform X5 [Pan paniscus]	Pan paniscus	688	688	97%	0.0	30.20%	1391	XP_024785515.1
cleavage and polyadenylation specificity factor subunit 1 isoform X3 [Pan paniscus]	Pan paniscus	622	622	88%	0.0	30.06%	1447	XP_034822808.1
cleavage and polyadenylation specific factor 1, 160kDa, isoform CRA_b [Homo sapiens]	Homo sapiens	261	261	24%	7e-76	36.58%	377	EAW82109.1
unnamed protein product [Homo sapiens]	Homo sapiens	237	237	20%	3e-68	38.77%	330	BAG57065.1
cleavage and polyadenylation specificity factor 160 kDa subunit [Pan troglodytes]	Pan troglodytes	214	214	16%	4e-61	41.43%	269	BAK62854.1

Figure S2. Circ-CPSF1 host gene Y76B12C.7a.1 is 32.22% identify with Homo sapiens CPSF1 at BLAST.
